# Evaluation of a modified sutureless technique for scleral fixation of one-piece posterior chamber intraocular lens: a retrospective study

**DOI:** 10.3389/fmed.2024.1503394

**Published:** 2025-01-13

**Authors:** Mohammadkarim Johari, Alireza Attar, Dorna Eghtedari, Seyed Ahmad Razavizadegan

**Affiliations:** Poostchi Ophthalmology Research Center, Department of Ophthalmology, School of Medicine, Shiraz University of Medical Sciences, Shiraz, Iran

**Keywords:** aphakia, posterior chamber intraocular lens, scleral pocket, transscleral suture fixation, Hoffman pocket

## Abstract

**Background:**

This study presents the one-year outcomes of a modified technique for transscleral suture fixation of a posterior chamber intraocular lens (PCIOL) in aphakic eyes.

**Methods:**

A retrospective chart review was conducted on 45 patients who underwent transscleral suture fixation of a foldable one-piece PCIOL through scleral pockets. Preoperative data and follow-up data for a minimum of 12 months were collected for all patients.

**Results:**

The mean preoperative and postoperative uncorrected distance visual acuity (UDVA) in LogMAR was 2.49 ± 0.54 and 0.54 ± 0.25, respectively (*p* < 0.01). The mean preoperative and postoperative best-corrected visual acuity (BCVA) in LogMAR was 0.52 ± 0.22 and 0.45 ± 0.24, respectively (*p* < 0.01). The mean endothelial cell loss was 110.46 ± 11.5 cells/mm^2^. Postoperative complications included transient corneal edema (17.7%), transient elevated intraocular pressure (22.2%), and non-significant vitreous hemorrhage (11.1%). No severe complications were observed.

**Conclusion:**

The modified technique provides stable PCIOL placement in aphakic eyes with long-term follow-up.

## 1 Introduction

Several surgical techniques are available for intraocular lens (IOL) implantation in the absence of capsular support (1). Among these, transscleral fixated posterior chamber intraocular lenses (PCIOLs) are particularly effective for correcting aphakia (2–5), especially in patients with corneal disease, iris tissue damage, angle abnormalities, or glaucoma. Positioned closest to the original lens location, PCIOLs offer several inherent advantages: they avoid contact with the corneal endothelium and trabecular meshwork, and they serve as a mechanical barrier between the vitreous cavity and the anterior chamber.

Techniques for transscleral fixation include *ab interno* methods (6, 7), where the suture is passed from inside the eye to the external surface, and *ab externo* methods (8, 9), where the suture is initially passed from the external surface. A common challenge across all transscleral fixation techniques is the need to bury, cover, or rotate the knot created for fixation. However, knot erosion and suture exposure remain potential complications, with reported incidence rates ranging from 6.7 to 73.0% (10, 11).

The Hoffman corneoscleral pocket technique for intraocular lens (IOL) fixation involves withdrawing the transscleral-conjunctival fixation sutures from within the pocket and then tying them (12). This method is preferred over the standard approach, which requires creating a conjunctival peritomy followed by a scleral flap to cover the fixation knot, allowing it to be tied under direct visualization.

The Hoffman pocket technique has been applied to various types of IOLs, particularly those with closed-loop haptics or specially designed eyelets on the haptics through which sutures can be passed for fixation (13–15). Additionally, some studies have evaluated the use of this technique for scleral fixation of secondary foldable three-piece PCIOLs (16, 17). In this study, we propose a modified technique for the scleral fixation of a secondary foldable one-piece PCIOL, demonstrating surgical success in 45 eyes. The long-term outcomes presented here assess the reliability and reproducibility of this novel technique.

## 2 Subjects and methods

A retrospective chart review was conducted on 45 eyes of 45 patients who underwent modified transscleral suture fixation of a one-piece PCIOL (Tecnis® ZCB00, Abbott Medical Optics, Santa Ana, CA) between February 2021 and March 2023. Patients were followed for at least one year postoperatively. The study adhered to the principles outlined in the Declaration of Helsinki and was approved by the institutional review board of Shiraz University of Medical Sciences. As the study was retrospective, the requirement for informed consent was waived.

Data collected included surgical indications, details of the primary surgery, the interval between the present and primary surgery, and relevant ocular and systemic history. A complete ophthalmic examination was conducted for all patients, including best-corrected visual acuity (BCVA), intraocular pressure (IOP), refraction, slit-lamp and dilated fundus examination. Axial length was measured using the IOL Master 700 in most cases or an A-scan ultrasound device (Alcon Ocuscan RXP), with IOL power calculated primarily using the Sanders-Retzlaff-Kraff Theoretical (SRK-T) formula, and the Kane formula in some instances. Corneal endothelial cell density (ECD) was measured preoperatively and at 3 months postoperatively. Postoperative complications were also recorded. Macular optical coherence tomography (OCT) (Spectralis OCT; Heidelberg Engineering, Heidelberg, Germany) was performed at 1, 3, and 6 months postoperatively to evaluate macular changes after surgery.

### 2.1 Surgical technique

All surgical procedures were performed by two vitreoretinal surgeons (MKJ and SAR) under general anesthesia. A marker pen was used to indicate the intended sites of the pockets on the sclera and conjunctiva. The two Hoffman corneoscleral pockets were created 180° apart, allowing flexibility in their placement, unlike previous methods, which were limited to specific positions like 12 and 6 o’clock. This approach provided better management options for patients with prior surgeries. First, two 3.0-mm-long clear corneal partial-thickness grooves were made using a 15° lance-tip blade (300 to 400 μm depth), just anterior to the conjunctival insertion at the limbus. The scleral pockets were then dissected posteriorly from the two opposing incisions using a crescent knife, extending approximately 3.0 mm posteriorly from the clear corneal incisions. Two separate round tip curved needles carrying polypropylene (Prolene) 8–0 sutures were tied to the IOL optical margins 180° apart, sutures were placed 3 mm away from the haptic-optic junction and 1 mm away from optical margin of IOLs, using a cow-hitch knot technique.

A standard three-port, transconjunctival 23-gauge pars plana vitrectomy (EVA system, Dutch Ophthalmic Research Center [DORC]) was performed. In cases of nucleus material or IOL drop, complete vitrectomies and IOL removal were done. In aphakic eyes filled with silicone oil, the oil was removed initially. If retinal tears or lattice degeneration were observed, prophylactic barrier laser treatment was performed. A paracentesis was made at 9 or 3 o’clock using a 3.2 mm sharp-tip keratome lance-tip blade. A slightly angled 27-gauge needle was then introduced into the eye, penetrating the conjunctiva and sclera at one side of the pocket beds, 1.0–1.5 mm posterior to the limbus, to apply the *ab externo* fixation technique. The needle was cut, and one end of the Prolene suture was grasped with retinal forceps, passed through the anterior chamber, and docked into the opening of the 27-gauge needle. The needle was then passed through the conjunctiva and the full thickness of the opposite side of the scleral pocket, and the other end of the Prolene suture was passed through the anterior chamber and docked into the 27-gauge needle in the superior Hoffman pocket, following the same procedure.

**Figure 1:**
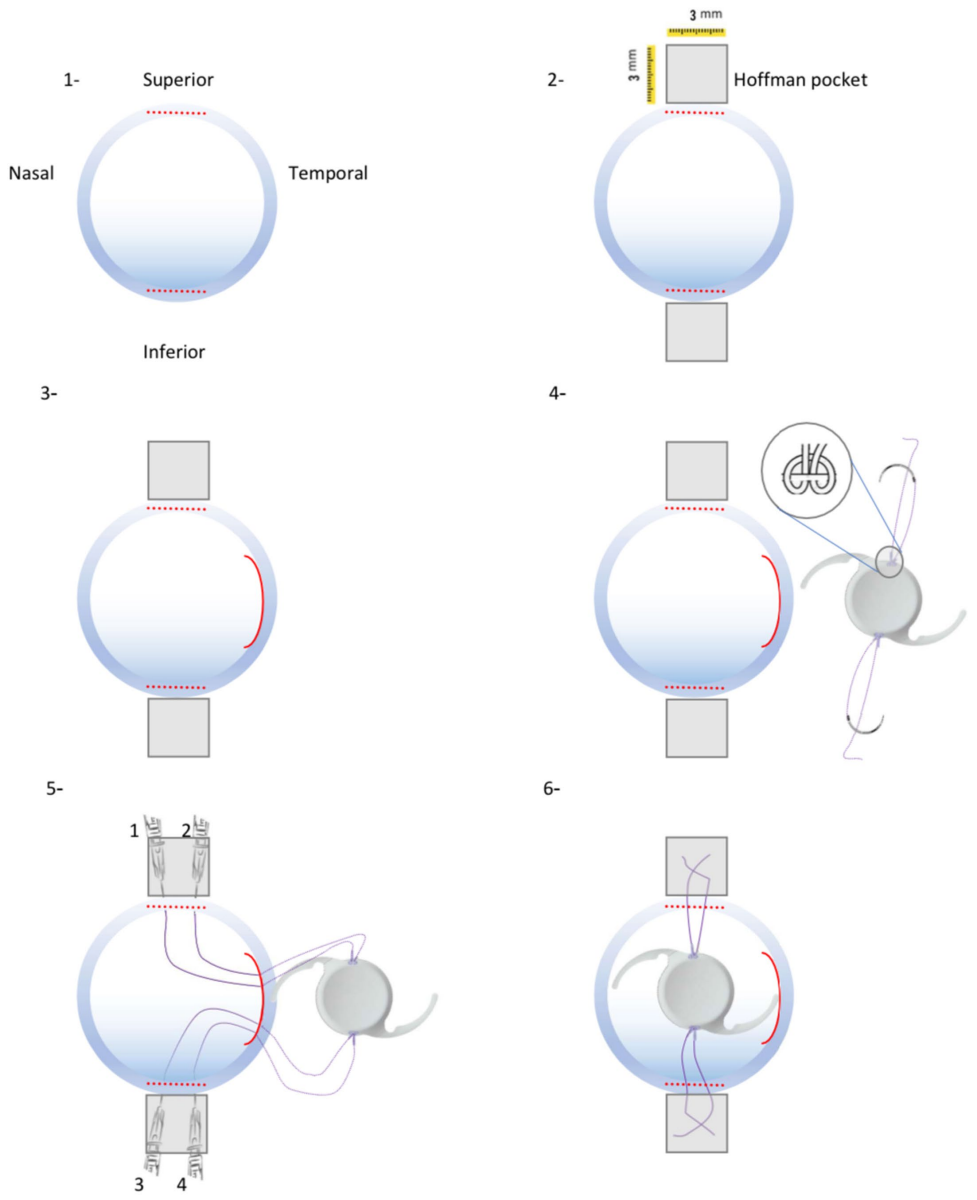
Schematic procedures of new technique for One-piece IOL fixation. (1) Two 3-mm wide limbus-parallel half-thickness corneal incisions were made in superior and inferior limbus. (2) Two 3 × 3 mm Hoffman scleral pockets were dissected. (3) 3.2-mm wide temporal corneal incision was made. (4) A single-armed suture was tied to both sides optical margin of the PCIOL. (5) Consequently 4 ends of Prolene 8–0 suture were passed through the temporal corneal incision and guided out of the eye from scleral pockets by a docking hollow needle. (6) Sutures were guided out of the eye from their corresponding scleral pocket beds and the PCIOL position was set by adjusting tension of each suture knot.

**Figure 2:**
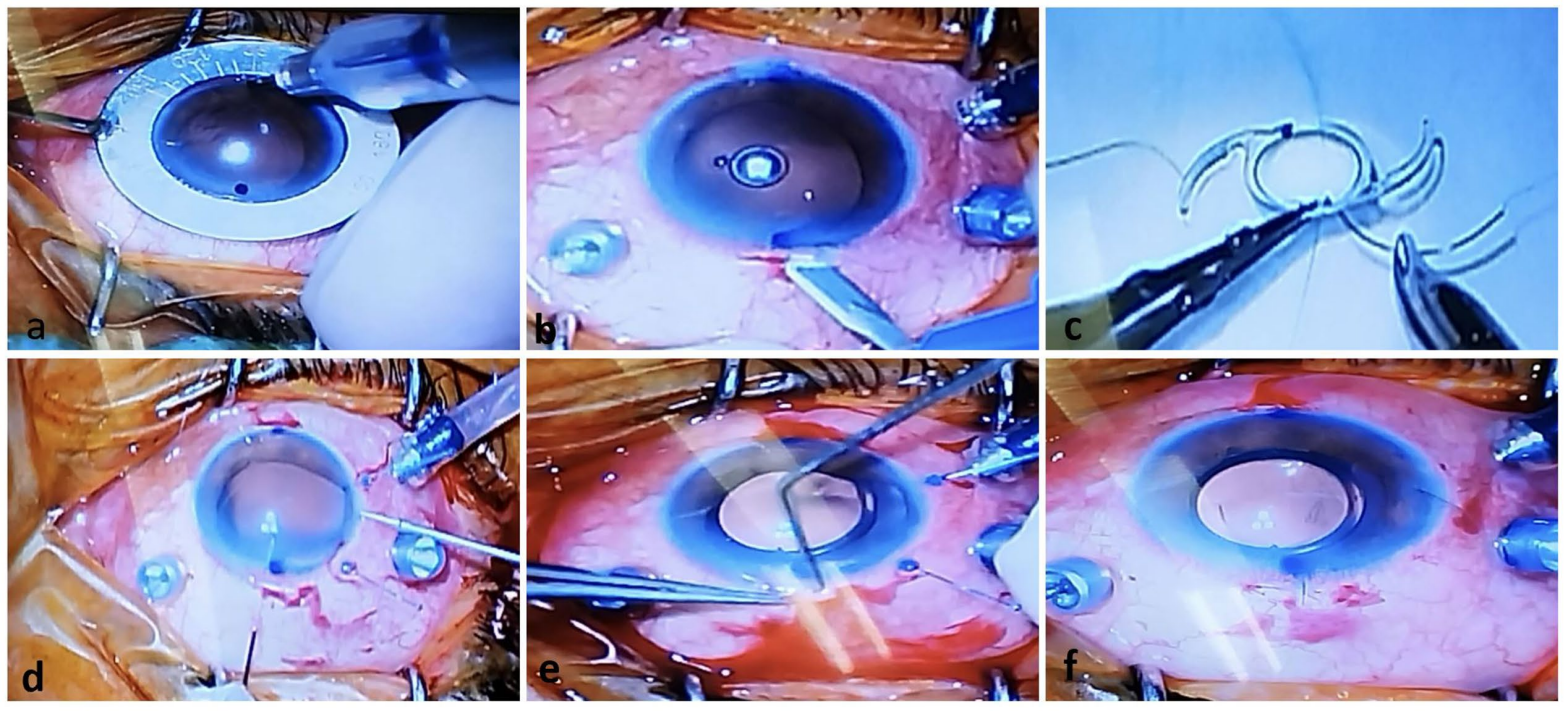
Operative photos of the new technique for One-piece IOL fixation. **(A)** Two-sided limbus was marked just against each other. **(B)** A Huffman pocket was made by crescent knife. **(C)** A single-armed sutures were tied to both marginal sides of optic of the PCIOL by cow-hitch knot. **(D)** A hollow needle was passed through the scleral pocket bed into the eye to dock and guide out the suture end coming from the temporal corneal incision by retinal forceps. **(E)** After IOL injection, sutures were take-out from pocket by sinskey hook. **(F)** The PCIOL position was set by adjusting tension of each suture knot.

This technique was repeated on the opposite side of the scleral pocket. The PCIOL was folded with McPherson suture-tying forceps, inserted through the temporal incision, and placed in the ciliary sulcus. The pairs of sutures on each side were retrieved externally through the scleral pockets using a Sinskey hook. After the PCIOL was positioned correctly behind the iris, the sutures at each pocket were knotted together. Tying the suture ends adjusted the IOL centrally, allowing the knot to be concealed as it slid under the protective roof of the scleral pocket (Figure 1: schematic images and Figure 2: intraoperative step-by-step images).

**Figure 3:**
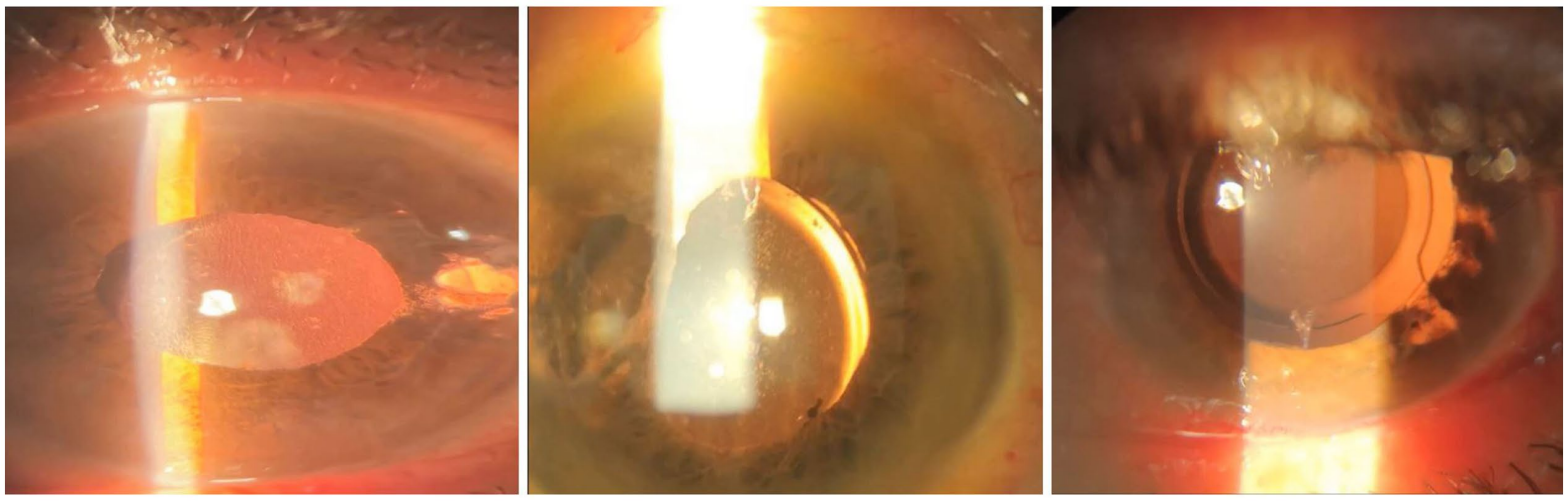
Images of three patients after 3-months follow-up who had scleral fixating IOL implantation.

There were no major intraoperative complications such as hyphema, vitreous hemorrhage, expulsive suprachoroidal hemorrhage, or IOL drop. Postoperatively, all patients were started on prophylactic antibiotic eye drops six times per day for 2 weeks, topical lubricating eye drops six times per day for 6 months, and topical prednisolone acetate 1% initially 10 times per day, with weekly tapering discontinued after 6 weeks (Figure 3: slit photos of three patients after 3-months follow-up).

### 2.2 Statistical analysis

Data were analyzed using descriptive and inferential statistical methods to evaluate the outcomes of the modified transscleral suture fixation technique. Continuous variables were summarized using means and standard deviations. Differences between preoperative and postoperative measurements were assessed using paired t-tests, with a significance level set at *p* < 0.05. The rate of endothelial cell loss (ECL) was calculated as the difference between preoperative and postoperative ECD. Postoperative complications, including corneal edema, elevated IOP, vitreous hemorrhage, and cystoid macular edema (CME), were reported as percentages of the total cohort.

## 3 Results

This technique was performed on 45 eyes of 45 patients (33 males and 12 females), with a mean age of 61.6 ± 16.05 years, ranging from 7 to 82 years. The mean follow-up time was 14.5 ± 5.7 months. Indications for surgery included prior aphakia or IOL drop due to complicated cataract surgery, spontaneous subluxation of the PCIOL (often due to pseudoexfoliation syndrome or iris coloboma), blunt or penetrating globe injuries resulting in aphakia, and aphakia following previous pars plana vitrectomy combined with silicone oil injection for rhegmatogenous retinal detachment (RRD) or endophthalmitis. Patient information is summarized in Table 1.

**Table 1:** Patients demographic date and causes of transscleral lens fixation.

Parameter	Number
Number of eyes	45
Male/Female	33/12
Mean age ± SD (y)	61.6 ± 16.05
Mean follow-up time ± SD (m)	14.5 ± 5.7
Cause of transscleral PCIOL fixation, *n* (%)	
Complicated cataract surgeries with IOL drop or with Aphakia	16 (35.8)
Spontaneous IOL dislocation or IOL drop	14 (31.7)
Traumatic IOL dislocation or IOL dropatic	8 (17.8)
Silicon filled eye with aphakia	6 (13.4)
PKP with artisan removal due to PBK	1 (2.2)

SD, standard deviation; PKP, Penetrating keratoplasty; PBK, Pseudophakic bullous keratopathy; IOL, intra ocular lens.

The mean postoperative LogMAR uncorrected visual acuity (UCVA) was significantly better than the preoperative LogMAR UDVA (0.54 ± 0.25 versus 2.49 ± 0.54, *p* < 0.01). The mean postoperative LogMAR best-corrected visual acuity (BCVA) also improved compared to the preoperative LogMAR BCVA (0.45 ± 0.24 versus 0.52 ± 0.22, p < 0.01). There was no significant difference between postoperative and preoperative intraocular pressure (IOP) (13.11 ± 2.87 versus 13.21 ± 2.51 mmHg, *p* > 0.05). The mean preoperative endothelial cell count (ECC) and postoperative ECC were 1,459 ± 547 and 1,358 ± 448 cells/mm^2^, respectively (*p* < 0.05), with a mean endothelial cell loss (ECL) of 110.46 ± 11.5 cells/mm^2^ (Table 2).

**Table 2:** Preoperative and postoperative functional results.

Parameters	Pre-operative	Post-operative	*p*-value
Mean LogMAR UCVA	2.49 ± 0.54	0.54 ± 0.25	0.001
Mean LogMAR BCVA	0.52 ± 0.22	0.45 ± 0.24	0.001
Mean IOP (mmHg)	13.21 ± 2.51	13.11 ± 2.87	0.43
Mean ECC (cells/mm2)	1,495 ± 547	1,385 ± 448	0.02
Mean SE(D)	9.50 ± 2.760	1.13 ± 0.54	0.001
Mean Astigmatism(D)	2.34 ± 0.75	1.94 ± 0.35	0.23

UCVA, Uncorrected Distance Visual Acuity; BCVA, Best-Corrected Visual Acuity; ECC, Endothelial Cell Count; SE, Spherical Equivalent.

Table 3 lists the postoperative complications. Central corneal edema was observed in eight patients (17.7%), and elevated IOP was noted in ten patients (22.2%) during the first week after surgery. Both complications resolved within 3 weeks with topical medication. Significant vitreous hemorrhage occurred in five patients (11.1%); one of these patients required a vitreous washout after 3 weeks due to non-clearing hemorrhage. Three patients with a history of traumatic endophthalmitis and silicone-filled eyes developed hypopyon postoperatively (6.6%), but they maintained a good red reflex and clear vitreous; the hypopyon resolved within 1 week with systemic and topical corticosteroids.

**Table 3:** Post scleral fixating operation complications.

Postoperative complications	*n* (%)
Transient corneal edema	8 (17.7)
Transient elevated IOP	10 (22.2)
Significant vitreous hemorrhage	5 (11.1)
Post operation hypopyon	3 (6.6)
Post operation hypotonia	3 (6.6)
Cystoid macular edema	3 (6.6)
Epiretinal membrane (ERM)	1 (2.2)

Three patients experienced decreased IOP the day after surgery (6.6%). A Seidel test was performed to rule out incision leakage, and the IOP normalized after 3 days with a contact bandage lens. Optical coherence tomography (OCT) revealed cystoid macular edema (CME) in three patients (6.6%) one month after surgery; all of these patients had undergone scleral fixation of the IOL due to complicated cataract surgery. CME resolved in all cases following a sub-tenon triamcinolone injection. One patient developed an epiretinal membrane (ERM) after 12 months, which was not clinically significant. Other complications such as retinal detachment, suprachoroidal hemorrhage, reverse pupillary block, and endophthalmitis were not observed. No patients experienced suture breakage or suture knot exposure.

## 4 Discussion

Managing aphakia in various situations can be challenging, with limited surgical options available. Anterior chamber intraocular lens (AC IOL) implantation is a reasonable option that avoids much of the procedural complexity associated with scleral fixation and may be more suitable in certain cases. However, difficulties can arise with AC IOL placement, particularly in younger patients or those with persistent inflammation, corneal decompensation, or glaucoma. Additionally, AC IOLs may not be appropriate for eyes without sufficient iris support. While there may not be a consensus on the best technique for placing a sutured IOL, there is agreement on the goals: reducing incision size, minimizing tilt, ensuring the longevity of the polypropylene suture, minimizing surgical time and complications, and ensuring applicability across different situations and IOL types.

In this study, we described the clinical outcomes of our modified technique for transscleral suture fixation of a PCIOL. All patients demonstrated improved visual outcomes after a mean follow-up period of 14.5 months. The complication rate was low, with no cases of suture breakage or suture exposure. No severe intraoperative or postoperative adverse events were observed during the study period. Dissecting the scleral pocket from a clear corneal incision using the Hoffman pocket technique avoids the need for conjunctival dissection, minimizes scleroconjunctival hemorrhage, and reduces the need for cautery. These factors may enhance postoperative comfort, reduce subsequent scar formation, and be advantageous for future glaucoma filtration surgery (16).

The duration of surgery was reduced by simplifying the scleral flap procedures. The average operative time for our patients was 40 ± 10 min, which may reduce the incidence of intraoperative complications. Suture degradation and breakage, particularly with 10–0 polypropylene sutures, can lead to IOL dislocation in scleral-fixated PCIOL, typically after 3 to 8 years (17). Our method employed an 8–0 polypropylene suture, which is less prone to biodegradation compared to 9–0 or 10–0 sutures. The sutures were tied to the IOL’s optics using a cow-hitch knot, eliminating free suture ends and reducing the risk of progressive loosening or slippage.

The Hoffman pocket technique has been used for scleral fixation with various types of IOLs, particularly those with closed-loop haptics or designed eyelets on the haptics, through which sutures can easily pass for fixation, or with 3-piece IOLs where the haptics are made of PMMA (12–15). The positioning of the scleral-fixated IOL is crucial for visual outcomes. Tilt greater than 15° can cause coma aberration, which cannot be corrected with spectacles, and optical decentration greater than 1.0 mm can cause radial astigmatism (18, 19). The number of points where the lens is supported by sutures is important for IOL stability. Two-point, four-point, and six-point fixation techniques using different types of IOLs have been reported (18–23). In these methods, the ends or loops of sutures are typically tied to the IOL haptics. In contrast, our technique involves tying the suture to the peripheral margin of the optic. In addition to providing four-point suture-scleral support, both haptics serves as “wings” for the IOL, placed in the sulcus to prevent dislocation or tilting, which is a key advantage of this technique. Although one of the limitations of our study was the lack of data from anterior segment OCT or UBM sonography to assess IOL tilt or decentration, the position and tilting of the IOLs could be estimated based on preoperative and postoperative astigmatism. In this study, the mean astigmatism of the eyes decreased after surgery compared to before surgery, which could serve as evidence for the proper positioning of the lens in the sulcus (2.34 ± 0.75, 1.94 ± 0.35 *p* = 0.23) (Table 2).

Haptic exteriorization techniques, first described by Yamani et al. and later by Baskaran and colleagues, involve needle-guided haptic insertion into a scleral tunnel for 3-piece IOL fixation (19, 21). However, due to the rigidity of PMMA haptics, there is a risk of disinsertion at the haptic-optic junction or IOL rotation within the scleral tunnel. Our method offers an effective, simple, safe, and minimally invasive approach for scleral suture fixation, applicable to any type of IOL in various situations.

No severe postoperative complications were observed. While there was a decrease in the mean endothelial cell count (ECC) after surgery (mean cell loss 110.46 ± 11.5 cells/mm^2^), none of the patients experienced prolonged postoperative corneal edema. We assessed the effect of this technique on macular structure using macular OCT. Only three patients developed cystoid macular edema, which resolved after a sub-tenon triamcinolone injection. Among all the patients, only one required reoperation due to non-clearing vitreous hemorrhage, which necessitated vitreous washing after 3 weeks. It is important to note that all of our patients had complex cases, and scleral fixation was performed in conjunction with pars plana vitrectomy.

## 5 Conclusion

Our modified technique for transscleral suture fixation of a one-piece PCIOL demonstrates promising results with improved visual outcomes and a low complication rate. The use of the Hoffman pocket technique and 8–0 polypropylene sutures provides a stable, effective, and minimally invasive solution for managing aphakia, even in complex cases. This approach reduces surgical time and minimizes complications, making it a reliable option for scleral fixation in a variety of clinical scenarios. However, it is important to note that due to the lack of anterior segment OCT or UBM imaging in this study, we were unable to directly assess IOL tilt or decentration. The reduction in astigmatism observed postoperatively suggests proper positioning of the IOL in the sulcus, but more comprehensive studies incorporating OCT or UBM are needed in the future to objectively confirm the position and alignment of the IOL achieved with this surgical technique.

## Data Availability

The datasets presented in this study can be found in online repositories. The names of the repository/repositories and accession number(s) can be found at: 09174872355.
